# Laparoscopic versus open hepatectomy for intrahepatic cholangiocarcinoma in patients aged 60 and older: a retrospective cohort study

**DOI:** 10.1186/s12957-022-02870-1

**Published:** 2022-12-13

**Authors:** Jianlei Wang, Wei Wang, Xiaolei Chen, Delin Ma, Gang Du, Tong Xia, Zhaochen Jiang, Bin Jin

**Affiliations:** 1 Department of Operative Surgery, Integration and Practice Center, Cheeloo College Department of Medicine, Shandong University, 44 Wenhuaxi Road, Jinan, 250000 Shandong China; 2 Department of Organ Transplantation, Qilu Hospital of Shandong University, Cheeloo College of Medicine, 107 Wenhuaxi Road, Jinan, 250000 Shandong China; 3 Center for Reproductive Medicine, Shandong University, 157 Jingliu Road, Jinan, 250000 Shandong China; 4 Department of Organ Transplantation, Qilu Hospital of Shandong University, 107 Wenhuaxi Road, Jinan, 250000 Shandong China

**Keywords:** Population aged 60 and older, Intrahepatic cholangiocarcinoma, Laparoscopic hepatectomy, Open hepatectomy, Prognosis

## Abstract

Objective laparoscopic surgical excision is the recommended treatment for liver cancers, yet its benefits in patients aged 60 and older remain poorly understood. Thus, this study evaluated the feasibility, safety, and clinical outcomes of laparoscopic hepatectomy for patients aged 60 and older with intrahepatic cholangiocarcinoma (ICC).

**Methods**

After screening, 107 patients who underwent hepatectomy for ICC were enrolled and grouped into either laparoscopic (LH) or open hepatectomy (OH) groups. Baseline characteristics, pathological findings, and long-term outcomes were compared between the two groups. Independent prognostic factors for overall survival (OS) and disease-free survival (DFS) were identified using univariate and multivariate analyses.

**Results**

Among baseline characteristics and pathological findings, only pre-operative albumin was higher in the LH group. The LH group had more favorable short-term outcomes such as incision length, level of postoperative total bilirubin, and length of postoperative stays than the OH group. The postoperative complication, lymph node dissection and R0 resection rate, and long-term outcomes including OS and DFS were not significantly different between the two groups. Cancer Antigen-19-9(CA-19-9) and pathological differentiation were independent prognostic factors for OS, whereas CA-19-9 and neutrophil count were independent prognostic factors for DFS.

**Conclusion**

LH is safe, reliable, and feasible for treatment of ICC patients aged 60 and older as it had better short-term clinical outcomes than OH and achieved long-term prognoses that were comparable to those of OH.

## Introduction

Liver cancers are highly malignant, often fatal, and have a global occurrence [1, 2]. They are classified, based on the source of tumor cells, into either hepatocellular carcinomas or cholangiocarcinoma, with the latter having more aggressiveness and poorer prognoses [2–4]. Anatomically, cholangiocarcinoma is divided into extrahepatic cholangiocarcinoma, hilar cholangiocarcinoma, and the more common intrahepatic cholangiocarcinoma (ICC). Most incidences of ICC are found in individuals aged 60 and older because the typical age for the onset of ICC is between 50 and 70 years [5]. Despite patients aged 60 and older having a low tolerance for intensive surgery, inferior cardiopulmonary function, and high risk of postoperative complications, complete surgical excision remains currently preferred treatment for all ICC patients [6, 7]. This surgical excision is performed via either conventional open hepatectomy (OH) or laparoscopic hepatectomy (LH). With the advance of modern medicine in terms of minimally invasive surgery and laparoscopic techniques in particular, LH has several advantages such as mild trauma, rapid recovery, lower intensity of pain, and short hospital stays [8, 9]. Nonetheless, LH may nonetheless lead to increased risk of cardiorespiratory and unpredictable problems [10]. Currently, the benefits of LH for ICC patients who were older remain poorly understood. Therefore, the present study examined short- and long-term outcomes of patients aged 60 and older with ICC who had undergone either OH or LH. The study also assessed potential risk factors for prognosis.

## Materials and methods

### Patient enrollment

Between January 2011 and October 2021, 107 ICC patients who had undergone hepatectomies at Qilu Hospital of Shandong University, Jinan, China, were enrolled, having fulfilled the following inclusion criteria: (1) patient with ICC confirmed by postoperative pathology, (2) patient underwent complete surgical tumor resection without macroscopic residual tumor (R0 or R1) and absence of distant metastasis, and (3) patient was monitored until date of mortality/last check-up. Surgical procedures were selected based on the following criteria: (1) According to whether it is minimally invasive surgery or not, the patients and their families chose the open or laparoscopic approach independently, (2) the surgeon evaluated the feasibility of laparoscopic surgery based on the location of the tumor, and (3) some patients who chose open surgery because of the difficulty of surgery were excluded (two patients who converted to open surgery due to intraoperative massive bleeding). In total, 29 patients who only underwent laparoscopic surgery during tumor resection were divided into the LH group, and 78 patients who underwent laparoscopic exploration or open surgery were divided into OH group. The present research represents a retrospective study, and the data used in this study were queried from Qilu Hospital of Shandong University databases, following approval by the hospital.

### Preoperative preparation

Both liver function tests and preoperative hematology examinations were conducted on all patients. Further, patients were regularly provided with support therapy and monitored using contrast-enhanced computed tomography (CT), magnetic resonance imaging (MRI), electrocardiograms (ECG), echocardiography, and specifically monitored via automatic 3D liver organ reconstruction that was used for on-demand volumetric analysis. Before surgery, patients fasted immediately prior to gastric tube insertion. Lastly, preoperative assessments and re-operative health education were required of all patients.

### Surgical technique

All patients were placed in the supine position after general anesthesia was administered. Routine surgical disinfection and draping were performed. For the LH group, pneumoperitoneum was established using the infraumbilical port, followed by insertion of three or four additional trocars. Before liver resection, tourniquets were prepared to possibly block the portal vein. Sterile gauze was used to expose the operative field and clean the surgical area. Standard laparoscopic instruments and an ultrasonic scalpel were used for surgical procedures such as partial hepatectomy, segment hepatectomy, and lobectomy whose election was determined by tumor location. If necessary, intraoperative ultrasonic and indocyanine green were used to locate tumors. For the major hepatectomy which involved 3 liver segments, and extended liver resection which involved more than 3 liver segments, such as left hemihepatectomy or right hemihepatectomy, we preferred the extrahepatic Glissonean approach. Minor liver resection was defined as the removal of one or two segments, the hepatic parenchymal transection approach was the preferred choice. For the OH group, traditional open hepatectomy was done with an anti-L-shaped incision of ca. 30cm. An ultrasonic scalpel (similar to that used in the LH group) was used to cut liver tissue, whereas absorbable and home-lock clamps were used to occlude the bile duct and vascular system. Bipolar electro-coagulation was regularly performed for hemostasis. Lymph node dissection (LND) is generally not performed in all patients at the center of our study, conversely, only performed in patients with enlarged lymph nodes on preoperative imaging or detected during intraoperative examination.

### Postoperative management

After surgery, all patients received professional postoperative management, nutritional support, rehydration, symptomatic support, and standard postoperative analgesic plans. On the first and third postoperative days, blood routine, coagulation tests, liver, and kidney functions and other indicators were examined. Additionally on the first postoperative days, gastric tubes were removed and on the fourth postoperative days, abdominal CT scans were done to assess patients’ intra-abdominal condition.

### Data collection and definitions

Patient’s medical records, which included demographic characteristics, comorbidities, preoperative and postoperative hematology and chemistries, intraoperative data, postoperative, and follow-up data, were collected retrospectively. Postoperative follow-ups were performed once every 3 months via telephone interviews. Overall survival (OS) was defined as the duration between surgery and death, and disease-free survival (DFS) was defined as the post-surgery duration that had no signs of disease. The 8th edition of AJCC/UICC TNM staging system and the Clavien-Dindo complication classification system were used to evaluate postoperative complications. According to the WHO latest principles of antibiotic classification, the antibiotic usage was divided into two categories: nonrestricted antibiotics and other antibiotic (restricted antibiotics and special class antibiotic).

### Statistical analysis

Categorical variables were presented as percentages and the statistical significance of their comparisons was assessed using either *χ*^2^ or Fisher’s exact tests. Continuous variables were expressed as medians/25th–75th percentiles/means/± standard deviations, and the statistical significance of their comparisons was assessed using either Student’s *t* or Mann–Whitney *U* tests. OS and DFS curves were plotted using Kaplan-Meier (KM) curves. Risk factors for OS and DFS were analyzed by Cox regression. All statistical analyses were done in SPSS Statistics V.25 (IBM SPSS Software) and/or R V.3.5.3, and a 0.05 alpha level was used for all tests.

## Results

### Patient characteristics

The baseline characteristics of patients in the two groups are summarized in the Table 1. Patients in LH and OH groups did not significantly differ (*p*>0.05) in terms of sex, age, body mass index (BMI), hypertension, diabetes, smoking, alcohol consumption, and hepatitis B virus infection. In addition, there were 11 (14.1%) and 5 (17.2%) patients with liver cirrhosis in the OH and the LH group, respectively, which was no significant differences (*p*=0.921). Moreover, no significant differences (*p*>0.05) in portal hypertension, ascites, preoperative antitumor treatment, and all preoperative blood tests except preoperative albumin (Alb) levels, which were significantly lower (*p*=0.039) in the OH compared to the LH group. However, the mean values of Alb for both groups were over the lower limit of normal, which is 40 g/L in our research center (Table 1).

**Table 1:** Comparison of the baseline characteristics between OH and LH groups

Variables	OH group (*n*=78)	LH group (*n*=29)	*P* value
Age, year	66.3±4.5	66.0±4.1	0.752
Sex, *n* (%)			0.123
Male	49 (62.8%)	13 (44.8%)	
Female	29 (37.2%)	16 (55.2%)	
BMI, kg/m^2^	23.8±3.7	24.8 ±3.8	0.200
Cirrhosis of the liver	11 (14.1%)	5 (17.2%)	0.921
Portal hypertension	2 (2.6%)	0 (0.0%)	1.000
Ascites	2 (2.6%)	0 (0.0%)	1.000
COPD	6 (7.7%)	1 (3.4%)	0.727
Diabetes	10 (12.8)	7 (24.1)	0.260
Hypertension	26 (33.3%)	12 (41.1%)	0.440
Coronary heart disease	4 (5.1%)	5 (17.2%)	0.106
History of smoking, *n* (%)	32 (41.0%)	11 (37.9%)	0.772
History of alcohol consumption, *n* (%)	28 (35.9%)	11 (37.9%)	0.846
Previous abdominal surgery, *n* (%)	18 (23.1%)	7 (24.1)	0.908
Hepatitis B virus infection, *n* (%)	13 (16.7%)	4 (13.8%)	0.949
Cholesterol, U/ml	4.9±1.6	4.4±0.83	0.155
PT	11.8±1.2	11.8±1.0	0.912
CA-19-9, U/ml	95.8 (18.5,527.3)	192.4 (22.1,904.3)	0.471
AFP, U/ml	3.2 (2.1,5.8)	3.6 (2.7,5.3)	0.651
CEA, U/ml	3.8 (1.8,23.6)	4.7 (2.8,17.0)	0.373
Mononuclear count, 10^9^/ml	0.8±1.5	0.5±0.16	0.297
Neutrophil count, 10^9^/ml	4.6±2.2	4.3±1.6	0.461
Lymphocyte count, 10^9^/ml	4.5±22.6	6.6±22.6	0.688
Platelet count, 10^9^/ml	231.4±81.1	232.2±73.7	0.961
HGB, g/L	131.9±18.7	136.8±18.5	0.237
ALT, U/L	19.5 (13.0,33.3)	18.0 (12.5,28.0)	0.360
AST, U/L	24.0 (18.0,36.5)	22.0 (18.0,27.5)	0.408
TBIL, umol/L	12.9 (8.5,21.0)	12.3 (10.1,14.6)	0.408
ALB, g/L	41.0±5.0	43.2±4.0	**0.039**
Preoperative antitumor treatment, *n* (%)	4 (5.1%)	2 (6.9%)	0.661

Data are presented as the mean with standard deviation (‾x±SD) or median with interquartile range (median [Q1, Q3]), or counts with percentages *n* (x%). Bold indicates statistical significance

*LH* laparoscopic hepatectomy, *OH*, open hepatectomy, *BMI* body mass index, *ASA* American Society of Anesthesiology, *CA-19-9* cancer antigen 19-9, *AFP* alpha fetoprotein, *CEA* carcinoembryonic antigen, *HGB* hemoglobin, *ALT* alanine aminotransferase, *AST* aspartate aminotransferase, *TBIL* total bilirubin, *ALB* albumin

### Pathological findings

A comparison of pathological findings between the OH and LH groups was as summarized in Table 2. Although the proportion of patients with lymph node metastasis was higher in the OH than the LH group (20.5 vs. 6.9%), this difference was not statistically significant (*p*=0.167). Other parameters without statistically significant differences in the two groups were pathological *differentiation, tumor number, tumor diameter, TNM* stage, surgical margin, microscopic microvascular invasion, microscopic perineural invasion, and satellite foci. It should be mentioned that no differences in major hepatectomy ratio were found between the two groups of our study (64.1 vs. 58.6%, *p*=0.167).

**Table 2:** Comparison of the pathologic findings between the OH and LH groups

Variables	OH group (*n*=78)	LH group (*n*=29)	*P* value
Tumor diameter, cm	5.8±3.0	5.0±2.1	0.177
Major hepatectomy, *n* (%)	50 (64.1%)	17 (58.6%)	0.602
Tumor number, *n* (%)			
Single	71 (91.0%)	26 (89.7%)	1.000
Multiple	7 (9.0%)	3 (10.3%)	
Pathological differentiation, *n* (%)			
Poorly differentiated	17 (21.8%)	3 (10.3%)	0.301
Moderately differentiated	47 (60.3%)	22 (75.9%)	
Well differentiated	14 (17.9%)	4 (13.8%)	
TNM stage, *n* (%)			
0/IA/IB/II	47 (60.3%)	19 (65.5%)	0.619
IIIA/IIIB/IV	31 (39.7%)	10 (34.5%)	
Lymphatic metastasis, *n* (%)	16 (20.5%)	2 (6.9%)	0.167
Surgical margin (R0), *n* (%)	72 (92.3%)	27 (93.1%)	1.000
Satellite foci, *n* (%)	7 (9.0%)	4 (13.8%)	0.710
Microscopic perineural invasion, *n* (%)	16 (20.5%)	7 (24.1%)	0.685
Microscopic microvascular invasion, *n* (%)	16 (20.5%)	6 (20.7%)	0.984

Data are presented as the mean with standard deviation (‾x±SD) or counts with percentages, *n* (*x*%)

### Surgical outcomes

Surgical outcomes for both OH and LH groups are summarized in Table 3. Incision lengths were obviously significantly shorter in the LH than OH group (5.7±2.3 vs. 20.1±1.5 *p*=0.000). Differences in operation time, albumin infusion, and intraoperative and postoperative transfusions were not statistically significant (*p*=0.907, *p*=0.070, *p*=0.185, and *p*=0.555, respectively). Similarly, R0 resection (92.3 vs. 93.1% *p*=1.000), liver door block (34.6 vs. 55.2% *p*=0.054) rates, and LND (47.4 vs. 27.6% *p*=0.064) rates were not significantly different between the two groups. In addition, there was no significant difference in the comparison of the number of lymph nodes between the groups (*p*=0.165). The LH group, had no intraperitoneal infections*, incision-*related *complications, delayed gastric emptying, and* bile *leakage nor* ICU admissions. The number of other postoperative complications—arrhythmology (0 vs. 3.4% *p*=0.271), peritoneal effusion (12.8 vs. 6.9% *p*=0.604), lung infection (14.1 vs. 3.4% *p*=0.227), myocardial infarction (0 vs. 6.9% *p*=0.072), and heart failure (2.6 vs. 10.3% *p*=0.122)—did not significantly differ between the two. Furthermore, grading of antibiotic was not different in both groups (*p*=1.000). Table 3 summarizes results of hematological examinations undertaken on the first postoperative day—total bilirubin levels (TBIL) were significantly higher in the OH than LH group (*p*=0.01). Plasma ALT and AST levels (median 297.0 and 261.5, respectively) in the OH group were higher than those (median 208.0 and 242, respectively) in the LH group (*p*=0.271 and *p*=0.446, respectively).

**Table 3:** Comparison of the surgical outcomes and follow-up outcomes between OH and LH groups

Variables	OH group (*n*=78)	LH group (*n*=29)	*P* value
Operation *time*, min	219.1±80.9	221.4±105.4	0.907
Albumin infusion, *n* (%)	68 (87.2%)	21 (72.4%)	0.070
*Intraoperative transfusion*, *n* (%)	19 (24.4%)	3 (10.3%)	0.185
*Postoperative transfusion*, *n* (%)	7 (7.7%)	4 (13.8%)	0.555
*Incision length*, cm	20.1±1.5	5.7±2.3	**0.000**
Lymph node dissection, *n* (%)	37 (47.4%)	8 (27.6%)	0.064
Lymph node yield			
0 nodes	41 (52.6%)	21 (72.4%)	0.165
1–5 nodes	21 (39.7%)	7 (24.1%)	
≥6 nodes	6 (7.7%)	1 (3.4%)	
Arrhythmology, *n* (%)	0 (0.0%)	1 (3.4%)	0.271
*Incision-*related *complications*	4 (5.1%)	0 (0.0%)	0.572
*Delayed gastric emptying*	1 (1.3%)	0 (0.0%)	1.000
Bile *leakage*	3 (3.8%)	0 (0.0%)	0.561
Peritoneal *effusion*	10 (12.8%)	2 (6.9%)	0.604
Intraperitoneal infection	5 (6.4%)	0 (0.0%)	0.320
Lung infection	11 (14.1%)	1 (3.4%)	0.227
Myocardial infarction	0 (0.0%)	2 (6.9%)	0.072
Heart failure	2 (2.6%)	3 (10.3%)	0.122
Liver door block, *n* (%)	27 (34.6%)	16 (55.2%)	0.054
ICU admission, *n* (%)	2 (2.6%)	0 (0.0%)	1.000
*Postoperative TBIL,* umol/L	31.1 (20.0, 38.4)	23.0 (16.9, 26.4)	**0.010**
*Postoperative ALT,* U/L	297.0 (139.8, 393.5)	208.0 (126.3, 10.5)	0.271
*Postoperative AST,* U/L	261.5 (156.0, 324.8)	242.0 (135.5, 302.5)	0.446
*Post-operative* stays, days	11.6±5.8	8.6±3.2	**0.009**
Grading of antibiotic			
Nonrestricted class antibiotic	25 (32.1%)	9 (31.0%)	1.000
Restricted class antibiotic and special class antibiotic	53 (67.9%)	20 (69.0%)	
Hospital cost, RMB(W)	7.7±3.2	7.2±1.7	0.502
Follow-up time, months	17.2±16.4	13.3±12.3	0.254
*30*-day *death*, *n* (%)	0 (0.0%)	0 (0.0%)	-
Total disease recurrence, *n* (%)	46 (59.0%)	11 (37.9%)	0.052
Postoperative antitumor treatment, *n* (%)	41 (89.1%)	9 (81.8%)	0.879
Repeated liver resection	11 (23.9%)	2 (18.2%)	0.994
RFA	4 (8.7%)	2 (18.2%)	0.326
Oncologic palliative therapy	19 (41.3%)	3 (27.3%)	0.607
Repeated liver resection and oncologic palliative therapy	3 (6.5%)	0 (0.0%)	1.000
RFA and oncologic palliative therapy	4 (8.7%)	2 (18.2%)	0.326
Sites of recurrence			
Hepatic	7 (15.2%)	1 (9.1%)	0.966
Extrahepatic	15 (32.6%)	4 (36.4%)	1.000
Hepatic and extrahepatic	24 (52.2%)	6 (54.5%)	0.887
Total death, *n* (%)	36 (46.2%)	9 (31.5%)	0.159

Data are presented as the mean with standard deviation (‾x±SD), or median with interquartile range (median [Q1, Q3]), or counts with percentages, *n* (x%)

Bold indicates statistical significance

*LH* laparoscopic hepatectomy, *OH* open hepatectomy, *RMB* Ren Min Bi, *RFA* radiofrequency ablation

It was found that postoperative hospital stays were significantly shorter in the LH than OH group (8.6 days vs. 11.6 days, *p*=0.009). However, hospital costs did not significantly differ between the two (7.7W RMB vs. 7.2W RMB, *p*=0.502). For both groups, no deaths were recorded within the first 30 days post-surgery.

### Long-term outcomes

**Fig. 1:**
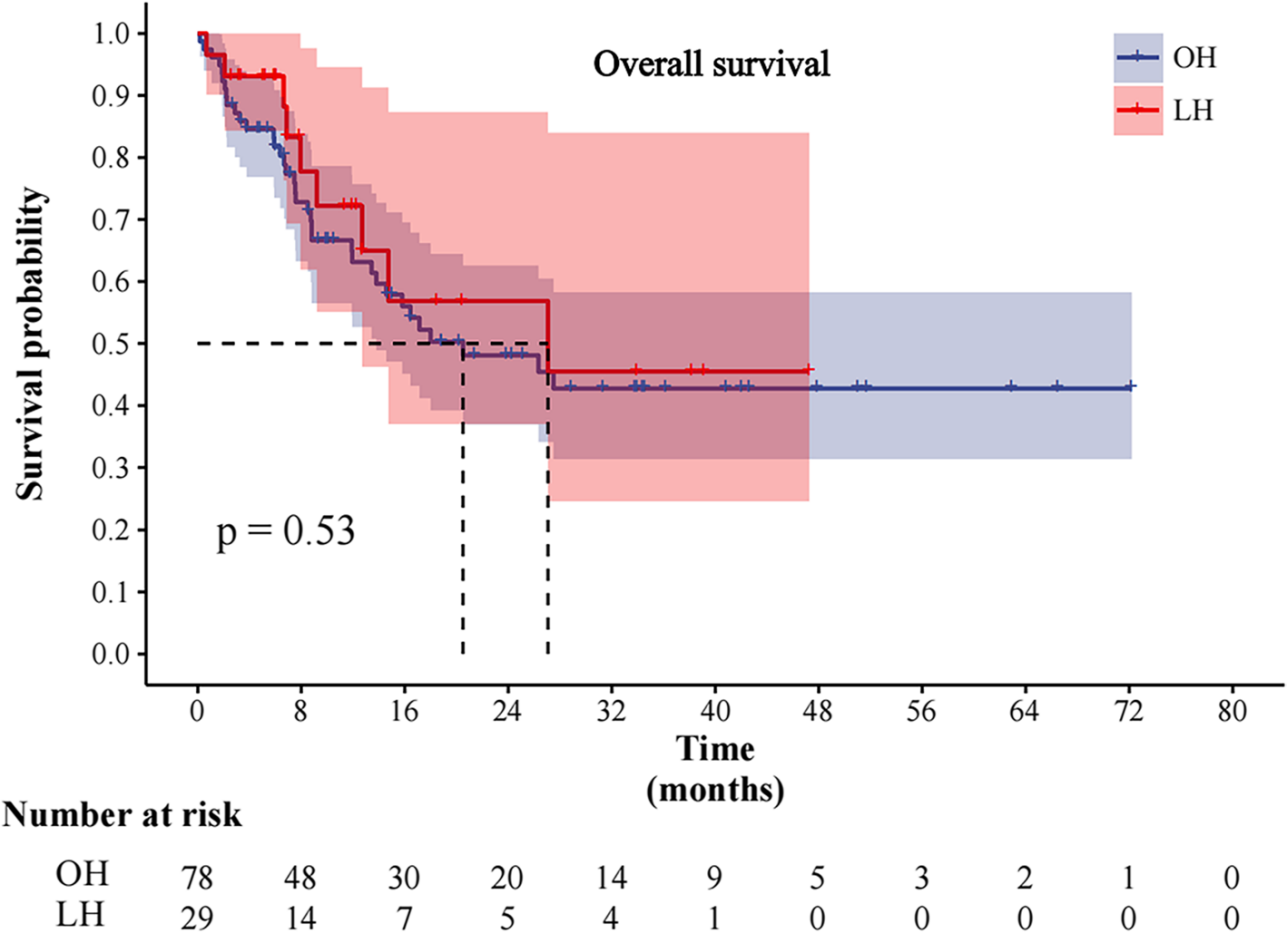
Comparison of overall survival between the two groups

**Fig. 2:**
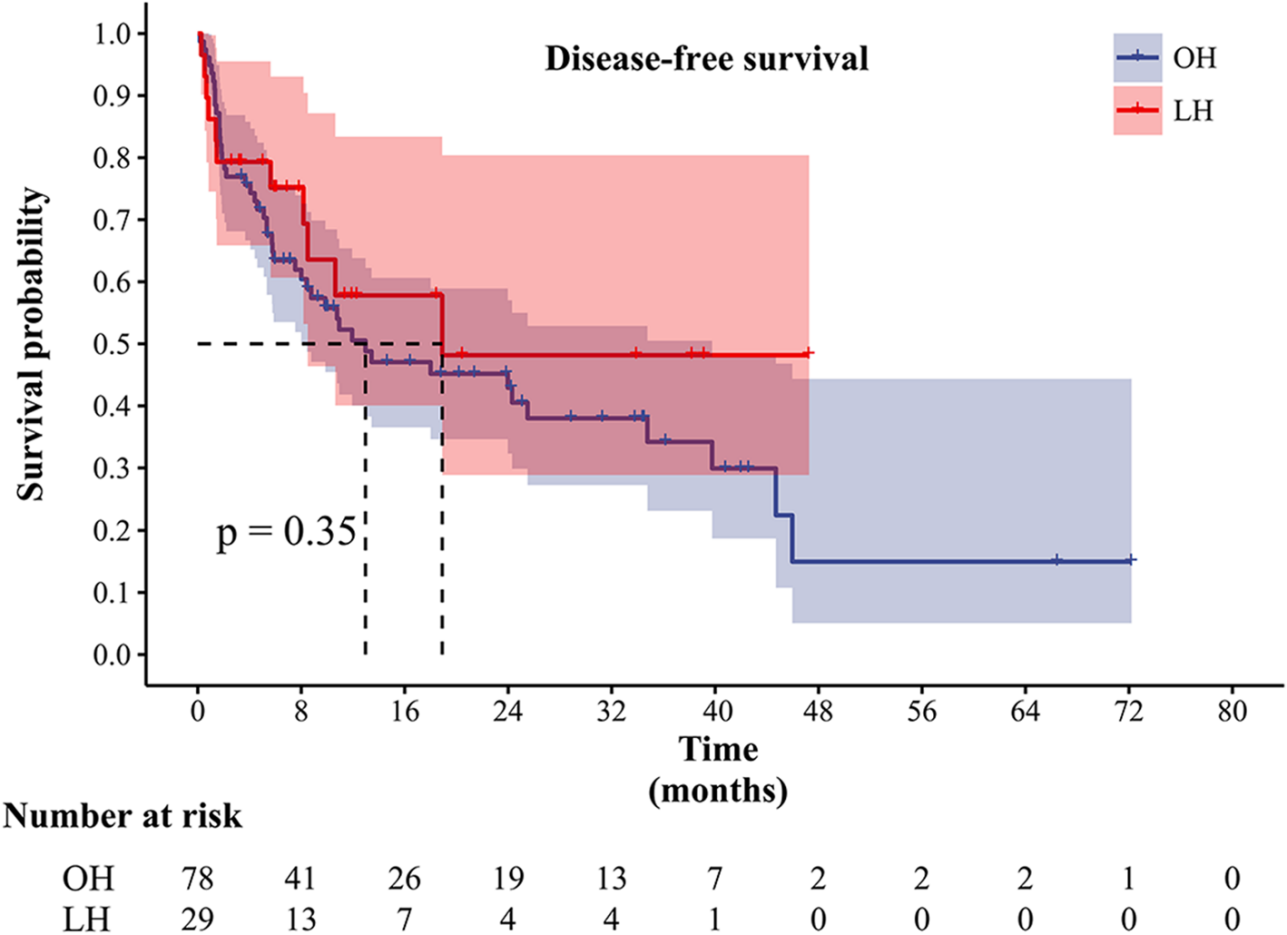
Comparison of disease-free survival between the two groups

Comparisons of long-term outcomes between the OH and LH groups are shown in Table 3 and Figs. 1 and 2. It was found that a higher proportion of patients relapsed (59.0 %) and died (46.2 %) in the OH group as compared with that in the LH group (37.9 % and 31.5 %, respectively). However, it was evident that the differences of relapse and death in the two groups were not statistically significant (*p* > 0.05). In patients who relapsed, the hepatic and extrahepatic recurrence (52.2% and 54.5%) was the main site of relapse in both groups, followed by hepatic recurrence (32.6% and 36.4%). The total number of patients who received treatment following recurrence are 41 and 9 patients in two groups, respectively (*p* > 0.05). Among the patients with recurrence in the two group, 4 patients (8.7%) and 2 patients (18.2%) received radiofrequency ablation (RFA), 11 patients (23.9%) and 2 patients (18.2%) received repeated liver resection, 19 patients (41.3%) and 3 patients (27.3%) received oncologic palliative therapy, 3 patients (6.5%) and 0 patients (0.0%) received repeated liver resection and oncologic palliative therapy, and 4 patients (8.7%) and 2 patients (18.2%) received RFA and oncologic palliative therapy.

The mean follow-up time was similar between the OH group and LH group (17.2±16.4 vs. 13.3±12.3, *p* = 0.254). The median OS and DFS for the OH group were 20.5 and 13.0 months, respectively, whereas for the LH group was 27.1 and 18.9 months, respectively (Figs. 1 and 2). These differences between the two groups were also not statistically significant (*p* = 0.53 and *p* = 0.35, respectively).

### Univariate and multivariate analyses of factors associated with OS and DFS

Results of univariate and multivariate analyses of variables for OS and DFS are summarized in Tables 4 and 5, respectively. CA-19-9, BMI, neutrophil count, TNM stage, platelet count, AST, pathological differentiation, lymphatic metastasis, satellite foci, and liver capsule invasion were independent prognostic factors for OS (*p*<0.05), and CA-19-9 (HR 1.002, 95% CI 1.001–1.003, *p*=0.000) and moderate differentiation (HR 0.344, 95% CI 0.159–0.744 *p*=0.007) were independent risk factors for OS. CA-19-9, TNM stage, neutrophil count, pathological differentiation, lymphatic metastasis, and liver capsule invasion were independent prognostic factors for DFS (*p*<0.05), and CA-19-9 (HR 1.001, 95% CI 1.000–1.002, *p*=0.006) and neutrophil count (HR 1.326, 95% CI 1.128–1.558, *p*=0.001) were independent risk factors for DFS.

**Table 4:** Univariate and multivariate analyses of factors associated with overall survival rates

Variable	Univariable cox regression analysis			Multivariable cox regression analysis		
	HR	95%CI	*P* value	HR	95%CI	*P* value
Sex male (vs. female)	1.209	0.662–2.210	0.537			
Age (years)	1.043	0.980–1.111	0.188			
Weight (kg)	0.977	0.953–1.001	0.063	0.993	0.930–1.061	0.845
BMI (kg/m^2^)	0.908	0.836–0.986	**0.022**	0.972	0.772–1.223	0.809
ASA score						
1	-	-	-			
2	0.450	0.108–1.882	0.274			
3	0.249	0.041–1.512	0.131			
Diabetes yes (vs. no)	0.720	0.304–1.704	0.455			
Hypertension yes (vs. no)	0.769	0.403–1.466	0.424			
Coronary heart disease yes (vs. no)	0.042	0.001–2.783	0.138			
History of smoking yes (vs. no)	1.002	0.554–1.812	0.995			
History of alcohol consumption yes (vs. no)	0.977	0.534–1.787	0.941			
Hepatitis B virus infection yes (vs. no)	0.696	0.310–1.562	0.380			
Liver cirrhosis, yes (vs. no)	0.481	0.189–1.221	0.124			
Ascites, yes (vs. no)	0.598	0.082–4.372	0.613			
CA-19-9 (U/ml)	1.002	1.001–1.003	**0.000**	1.002	1.001–1.003	**0.000**
AFP (U/ml)	1.004	0.999–1.010	0.105			
CEA (U/ml)	1.002	0.999–1.005	0.169			
Neutrophil count (10^9^/L)	1.334	1.145–1.554	**0.000**	1.207	0.993–1.467	0.059
Lymphocyte count (10^9^/L)	0.930	0.765–1.130	0.465			
Platelet count (10^9^/L)	1.004	1.000–1.008	**0.042**	1.001	0.996–1.005	0.769
HGB (g/L)	0.987	0.973–1.001	0.062	0.991	0.970–1.012	0.389
ALT (U/L)	1.000	0.995–1.004	0.819			
AST (U/L)	1.006	1.000–1.012	**0.035**	1.003	0.996–1.010	0.432
TBIL (umol/L)	1.001	0.997–1.005	0.546			
ALB (g/L)	0.970	0.922–1.022	0.251			
Tumor diameter (cm)	1.070	0.977–1.172	0.143			
Tumor number multiple (vs. single)	1.662	0.582–4.747	0.342			
Pathological differentiation						
Poorly differentiated	-	-	-			
Moderately differentiated	0.417	0.213–0.816	**0.011**	0.344	0.159–0.744	**0.007**
Well differentiated	0.502	0.200–1.259	0.142	0.840	0.317–2.548	0.926
TNM stage >II (vs. ≤II)	3.912	2.138–7.156	**0.000**	2.337	0.850–6.428	0.100
Lymphatic metastasis, yes (vs. no)	4.331	2.225–8.431	**0.000**	1.804	0.683–4.765	0.234
Satellite foci yes (vs. no)	2.535	1.122–5.725	**0.025**	0.414	0.136–1.261	0.121
Microscopic microvascular invasion yes (vs. no)	1.574	0.774–3.200	0.210			
Liver capsule invasion yes (vs. no)	1.929	1.020–3.646	**0.043**	1.244	0.464–3.337	0.664
Microscopic perineural invasion yes (vs. no)	1.579	0.775–3.219	0.208			
Lymph node dissection yes (vs. no)	1.420	0.791–2.550	0.240			
Number of lymph node dissection						
0						
1–5	1.480	0.805–2.719	0.207			
≥6	1.132	0.339–3.775	0.840			
Operative time (min)	1.002	0.999–1.005	0.267			
Surgical margin R1 (vs. R0)	0.936	0.288–3.045	0.913			
LH (vs. OH)	0.789	0.380–1.642	0.527			

Bold indicates statistical significance

*BMI* body mass index, *ASA* American Society of Anesthesiology, *CA-19-9* cancer antigen 19-9, *AFP* alpha fetoprotein, *CEA* carcinoembryonic antigen, *HGB* hemoglobin, *ALT* alanine aminotransferase, *AST* aspartate aminotransferase, *TBIL* total bilirubin, *ALB* albumin, *LH* laparoscopic hepatectomy, *OH* open hepatectomy

**Table 5:** Univariate and multivariate analyses of factors associated with disease-free survival rates

Variable	Univariable cox regression analysis			Multivariable cox regression analysis		
	HR	95%CI	*P* value	HR	95%CI	*P* value
Sex male (vs. female)	1.217	0.710–2.086	0.475			
Age (years)	1.008	0.951–1.069	0.783			
Weight(kg)	0.985	0.964–1.006	0.158			
BMI (kg/m^2^)	0.937	0.872–1.007	0.077	1.005	0.926–1.090	0.914
ASA score						
1	-	-	-			
2	0.566	0.136–2.347	0.433			
3	0.374	0.072–1.956	0.244			
Diabetes yes (vs. no)	0.812	0.383–1.721	0.587			
Hypertension yes (vs. no)	0.732	0.410–1.306	0.290			
History of smoking yes (vs. no)	1.025	0.605–1.737	0.928			
History of alcohol consumption yes (vs. no)	0.876	0.507–1.012	0.634			
Hepatitis B virus infection yes (vs. no)	1.075	0.553–2.088	0.831			
Liver cirrhosis, yes (vs. no)	0.559	0.253–1.236	0.151			
Ascites, yes (vs. no)	0.545	0.075–3.963	0.548			
CA-199 (U/ml)	1.001	1.001–1.002	**0.000**	1.001	1.000–1.002	**0.006**
AFP (U/ml)	1.003	0.998–1.009	0.195			
CEA (U/ml)	1.001	0.998–1.004	0.503			
Neutrophil count (10^9^/L)	1.360	1.183–1.564	**0.000**	1.326	1.128–1.558	**0.001**
Lymphocyte count (10^9^/L)	0.933	0.792–1.098	0.402			
Platelet count (10^9^/L)	1.002	0.999–1.006	0.213			
HGB (g/L)	0.991	0.978–1.005	0.203			
ALT (U/L)	1.000	0.997–1.003	0.954			
AST (U/L)	1.003	0.997–1.009	0.286			
TBIL (umol/L)	1.001	0.997–1.005	0.722			
ALB (g/L)	0.978	0.933–1.025	0.348			
Tumor diameter (cm)	1.060	0.978–1.149	0.155			
Tumor number multiple (vs. single)	1.372	0.540–3.489	0.507			
Pathological differentiation						
Poorly differentiated	-	-	-			
Moderately differentiated	0.534	0.290–0.982	**0.044**	0.722	0.378–1.380	0.324
Well differentiated	0.470	0.199–1.111	0.085	1.033	0.409–2.611	0.945
TNM stage >II (vs. ≤II)	3.463	2.019–5.940	**0.000**	2.224	0.837–5.913	0.109
Lymphatic metastasis, yes (vs. no)	3.972	2.094–7.535	**0.000**	1.585	0.637–3.943	0.322
Satellite foci yes (vs. no)	1.713	0.765–3.838	0.191			
Microscopic microvascular invasion yes (vs. no)	1.851	0.998–3.435	0.051	1.509	0.719–3.166	0.277
Liver capsule, invasion yes (vs. no)	1.978	1.113–3.515	**0.020**	1.286	0.558–2.965	0.555
Microscopic perineural invasion yes (vs. no)	1.402	0.715–2.749	0.325			
Lymph node dissection yes (vs. no)	1.242	0.737–2.093	0.415			
Number of lymph node dissection						
0						
1–5	1.229	0.711–2.125	0.461			
≥6	1. 320	0.464–3.756	0.603			
Operative time (min)	1.002	0.999–1.005	0.238			
Surgical margin R1 (vs. R0)	1.029	0.370–2.861	0.957			
LH (vs. OH)	0.734	0.379–1.418	0.357			

Bold indicates statistical significance

*BMI* body mass index, *ASA* American Society of Anesthesiology, *CA-19-9* cancer antigen 19-9, *AFP* alpha fetoprotein, *CEA* carcinoembryonic antigen, *HGB* hemoglobin, *ALT* alanine aminotransferase, *AST* aspartate aminotransferase, *TBIL* total bilirubin, *ALB* albumin, *LH* laparoscopic hepatectomy, *OH* open hepatectomy

## Discussion

ICC is the second most common primary malignant hepatic tumor [11] and most remain undiagnosed at early stages due to lack of typical distinct symptoms and have poor prognoses [3, 6]. The same phenomenon was observed in our study, where patients with relapse had a high mortality rate in both groups (36/46 and 9/11, respectively). Moreover, these tumors remain most neglected in the patients aged 60 and older, due to low awareness of the illness.

Surgical resection, especially R0, is the main treatment for ICC patients despite its considerable risk of recurrence [3, 6, 7]. In this study, most patients in both groups had R0 resection, indicating that laparoscopic surgery was now relatively feasible for ICC treatment. Rather than only in our research, many research centers had demonstrated the same attitude [12–15]. Relatedly, LH, when compared to OH for ICC treatment, had a higher R0 resection, more favorable short-term outcomes and lower LND rates [8, 9, 14, 15]. Furthermore, it was found that the LND rates in this study were lower in the LH as compared with the rates in OH group (Table 3), but the differences were not statistically significant.

Lymph node metastasis (LNM) had been established as a critical risk factor for prognosis in ICC [16], and the controversy of lymph node dissection for ICC had been attracting great attention in recent years. Advocates of routine LND in ICC patients suggest that LND not only prolongs OS and DFS but also allows for accurate lymph node staging, which can help in determining the patient’s prognosis and developing subsequent adjuvant treatment plans [17, 18]. While opponents argue that LND fails to improve OS or DFS in such patients and was responsible for the increased risk of postoperative complications [19]. Therefore, we performed the cox regression analysis to explore the clinical significance of LND in our experiments. The results revealed that both LND and the number of LND may not be an independent prognostic factor for OS and DFS. This may suggest that LND does not affect the prognosis and outcome in patients aged 60 and older with ICC.

Mean tumor diameters were larger in the OH than LH group, but this difference was not statistically significant. This difference may be because, logically and ethically, conventional open surgery was recommended for patients with large tumors. However, the rate of major hepatectomy via laparoscopic in LH group is comparable to OH group in our research center, which benefitted greatly from advances in laparoscopic instruments and techniques and indicated that laparoscopic major hepatectomy is also can performed. The significant differences in incision length and the duration of postoperative stays between the two groups (Table 3) corroborated previous studies [8, 12]. An advantage of shorter incision lengths in LH when compared to OH is prevention of possible complications and pain reduction. Indeed, the LH group had a zero incidence of incision*-*related complications. Postoperative infection, particularly lung infection in elderly patients, is an increasingly common clinical complication [20–22]. It is evident that no significant distinction was indicated in antibiotic usage. In this case, the rates of lung and intraperitoneal infections were lower in the LH than OH group. Therefore, a worthwhile research study would be to explore how laparoscopic surgery can be used to reduce postoperative infections in the elderly.

Owing to the dismal prognosis of ICC, it is urgent to find prognostic indicators to improve the prognosis. Perioperative level of Alb is reported as a possible independent prognostic factor for ICC [23–25]. In our present study, the preoperative levels of Alb significantly differed between the two groups (Table 1). However, we tentatively suggest that Alb played little role for the long-term outcomes in the study. There are two explanations. First, mean Alb levels for both groups not only slightly differed but also were over the lower limit of normal (Table 1). Second, a substantial proportion of patients lacked sufficient c-reactive protein (CRP) levels; thus, CRP/Alb ratios could not be calculated [24–26]. The total bilirubin level (TBIL) is a significant index of liver function [27–30], and thus, TBIL was monitored and high TBIL was promptly treated. Additionally, TBIL was an important independent prognosis predictor for ICC patients [31–33] and was significantly higher in the OH than LH group on the first postoperative blood tests (Table 3). However, TBIL did not differ between the two before operations, further demonstrating laparoscopic in lieu of open surgery as a means to prevent high TBIL. Body mass index (BMI), which is calculated from weight and height, is highly correlated with obesity. Obesity is a major concern that is correlated with increasing risks of cancers, including ICC [34, 35]. Likewise, the BMI is also known to be associated with the prognosis of ICC [36]. In the present study, we demonstrated that BMI was an independent prognostic factor for ICC (Tables 4). The ratio of the neutrophil count and other inflammatory biomarkers can predicate the prognosis of an ICC patient [37, 38]. Neutrophil count also affect prognoses and we also demonstrated this (Tables 4 and 5). The understanding of those risk factors and protective factors associated with prognosis is important to guide future interventions directed at improving long-term prognosis for of patients with ICC.

Research on treatments that ensure long-term survival of patients is high relevant for clinical cancer. Today, surgery-based comprehensive therapy combined with chemotherapy and molecular targeted therapy is the recommended basic route. In terms of surgery-based comprehensive therapy, this study demonstrated that ICC patients who underwent LH achieved long-term outcomes comparable to those of patients who underwent OH, and this corroborated previous studies [8, 12, 13]. Taken together, LH was a safe and feasible alternative treatment for ICC patients aged 60 and older, which achieved short and long-term outcomes that were comparable and even superior to those of OH.

There are two limitations in the study should be point out. Firstly, it was a retrospective analysis. Second, it had a small sample size (*n*=107) due to insufficient medical records for a large proportion of patients who were from other research centers. The latter especially limited the study as the robustness of the employed methods increases with the amount of systematic information and sample size.

## Conclusion

In conclusion, LH was safe, reliable, and feasible for the treatment of ICC patients aged 60 and older. We also found that the rate of major hepatectomy and LND in the LH group can achieve the same effects as the OH group. Furthermore, patients in the LH group had better short-term clinical outcomes as compared with that in OH whereas the long-term prognoses of patients in the two group were statistically comparable to each other.

## Data Availability

The datasets used and/or analyzed during the current study are available from the corresponding author on reasonable request.

## Abbreviations

ICC: Intrahepatic cholangiocarcinoma
LH: Laparoscopic hepatectomy
OH: Open hepatectomy
CT: Computed tomography
MRI: Magnetic resonance imaging
RFA: Radiofrequency ablation
ICU: Intensive care unit
BMI: Body mass index
TBIL: Total bilirubin level
AST: Aspartate aminotransferase
ALT: Alanine aminotransferase
OS: Overall survival
DFS: Disease-free survival
HR: Hazard ratio
CI: Confidence interval
LND: Lymph node dissection
